# Supplementary material for: Learning curve and surgical outcome of robotic assisted colorectal surgery with ERAS program
Source: Sci Rep. 2022 Nov 29;12:20566. doi: 10.1038/s41598-022-24665-w (PMC9709162; doi:10.1038/s41598-022-24665-w)
Supplement: Supplementary file 1 — Supplementary Information 1. [file 41598_2022_24665_MOESM1_ESM.zip › LSC Raw data-Ñ[▒K/119/H_ñw╝╨░O▒Kñσ.pdf]

正式摘要(電子簽章：000000211848482)

W52 / W52 ☐ 英文標題(注意:無英文姓名-請補病人基本檔!) ☐【讀取】憑證

# 臺中榮民總醫院
## 出院病歷摘要

轉入號 : 轉出號 :

[redacted]

| | |
|---|---|
| **(8)**流水編號 | |
| **(9)**入院日期 | 110/03/03 (2021) 直腸外科 W52 - 152 |
| **(10)**轉科日期 | |
| **(11)**出院日期 | 110/03/08 (2021) 住院天數計 5 日 |

## 診斷

- 入院 :1. Suspect colon cancer, asceniding colon
  [PH]
  1. CAD s/p stent in 2018
  2. L spine spondylothesis s/p spinal surgery in 2016
  3. HTN under medication
- 出院 :1. Colon cancer, asceniding colon, s/p laparoscopic right hemicolonectomy and anastomosis on 2021/03/04, pending pathology
  [PH]
  1. CAD s/p stent in 2018
  2. L spine spondylothesis s/p spinal surgery in 2016
  3. HTN under medication

## 主訴

- stool OB (+)

## 病史

- This 76 y/o female with a hx of
  1. CAD s/p stent in 2018
  2. L spine spondylothesis s/p spinal surgery in 2016
  3. HTN under medication
  This time, stool OB was noted at regular physical examination in 2021/01 at 李綜合H. There was no abdominal pain , contipation, diahrrea, difficulty in defecation nor bloody stool was noted. There was also no general weakness, dizziness or abdominal pain. Colonwscope was thus done at 李綜合H and mass over ascending colon was noted. Operation was suggested but the patient hesitated. She then came to TCVGH for second opionion. Under the impression of suspect malignant neoplasm at ascending colon, she was admitted for further evaluation and management.

## 體檢發現

- Anal inspection:
  Recto digital examination:
  Sigmoidoscopy:
  Anoscopy:
  Abdomen:
  - General appearance :
    - easy looking , conscious clear , without cardiopulmonary distress
    - Vital signs自動帶入date 體溫(BT) 脈搏(P) 呼吸(R) 血壓(BP) 身高(BH) 體重(BW) BMI
      20210303 35.9℃ 77次/分 20次/分 141/79mmHg 154.5cm 62.7kg 26.3
    - Vital signs: B.P. 141/79 mmHg , P.R. 77 /min , R.R. 20 /min , B.T. 35.9 ℃
  - HEENT :
    - Eyes: no anemic conjunctiva, no icteric sclera, normal EOM
    - ENT: no otorrhea, no rhinorrhea, no erythematous mucosa, no injected tonsil, no deformity
  - Neck: supple, freely movable, trachea midline,no deformity, no engorged jugular

- vein , no palpable thyroid or lymph node enlargement
- Chest:
  - Inspection: Grossly normal, symmetric and fully expansion, no accessory muscle used, no operation scar, no spider angiomata, no gynecomastia
  - Palpation: no palpable mass or nodule, no subcutaneous emphysema, no tenderness
  - Ausculation: clear breathing sound
- Heart:
  - Palpation: no thrill, no heave
  - Percussion: normal heart size
  - Ausculation: regular heart beat, normal S1, S2, no gallop, no murmur
- Extremities: No deformity, full ranged of motion without limitation, no clubbing finger or nail cyanosis, no pitting edema, no ulcer or pigmentation, no obvious skin lesion, no varicose vein, no bone, joint or muscle tenderness

### CRS CONDITION

- Karnofsky performance status: (100) Normal, no complaints, no evidence of disease
- PE of Abdomen:
- CEA: pending ng/ml
- Imaging Studies:
- Colonoscope report: Sedative colonoscopy up to cecum, excellent preparation. One ulcerative tumor near to IC valve. One small polyp at A-colon.
- Figures:
  1. colon & rectum

## 手術日期及方法

- 手術日期及方法20210304 Laparoscopic right colectomy and anastomosis
  20210303 Colonoscopy

## 住院治療經過

- After admission, no contraindication was noted for laparoscopic right hemicolonectomy. Colonscope was first done and showed one ulcerative tumor near to IC valve and one small polyp at A-colon. Operation was then performed smoothly on 2021/03/04. Under ERAS protocol, she started BLAND I diet on the next day. Foley was first removed on the same day but re-on on 03/06 due to urinary retention. PCA was off on 03/06 and foley was again renoved on 03/07. No urinary retention was noted and she started BLAND II diet. Flatus was noted on 03/07. Surgical wound was clean and no discharge. Since the patient was under a relatively stable condition, she was then discharged on 03/08. OPD f/u was also arranged.

## 合併症

## 一般檢查

- 累積報告 :

| DATE | TIME | ALB | TP | BIL.T | BIL.D | ALKP | AST | ALT | LDH | CHOL | TG | HDL-C | LDL | UA | NH3 | AMY |
|---|---|---|---|---|---|---|---|---|---|---|---|---|---|---|---|---|
| 1100303 | 1902 | 3.8 | 6.2 | 1.00 | 0.17 | 91 | 21 | 18 | 169 | 186 | 122 | 53 | 108 | 5.6 | | |
| 1100304 | 1854 | 3.4 | | 0.8 | 0.2 | | 21 | 14 | | | | | | | | |

| DATE | TIME | WBC | RBC | HGB | HCT | MCV | PLT | BLST | NEUT | BAND | SEG | LYM | MON | EOS | BAS |
|---|---|---|---|---|---|---|---|---|---|---|---|---|---|---|---|
| 1100303 | 1902 | 5930 | 4.09 | 11.8 | 35.9 | 87.8 | 279 | | 56.7 | | | 34.2 | 6.7 | 1.7 | 0.7 |
| 1100304 | 1852 | 9500 | 4.03 | 11.6 | 35.8 | 88.8 | 274 | | 80.5 | | | 12.7 | 6.4 | 0.2 | 0.2 |

| DATE | TIME | NA | K | CL | CA | BUN | CREAT | Mg | P | CRP | ESR | PCT | LIPASE | LACTATE |
|---|---|---|---|---|---|---|---|---|---|---|---|---|---|---|
| 1100303 | 1902 | 142 | 3.4 | 104 | 8.2 | 14 | 0.83 | | | 0.621 | | | | |
| 1100304 | 1854 | 141 | 3.6 | | | 9 | 0.77 | | | | | | | |

## 特殊檢查

## 病理報告

- pending

## 其它

## 出院時情況

- 3.改門診治療

## 出院指示

- | 藥名 | 劑量 | 劑量單位 | 用法 | 頻次 | 數量 | 數量單位 | 現況 | 天數 | 單位 |
  |---|---|---|---|---|---|---|---|---|---|
  | Ultracet tab | 1 | TAB | PO | Q6HPRN | 20 | TAB | TH | 5 | 天 |
  | Cap Trand 250mg | 1 | CAP | PO | BIDPRN | 10 | CAP | TH | 5 | 天 |

## 出院計劃

主治醫師 2062F 蔣鋒帆 / 住院醫師 9757K 翁明謙

正式摘要**(**電子簽章：**000000211848482)**

W52 / W52

doctorRank:B
